# Characteristics of early-onset hematotoxicity of sunitinib in Japanese patients with renal cell carcinoma

**DOI:** 10.1186/s12885-017-3205-9

**Published:** 2017-03-23

**Authors:** Renpei Kato, Yoichiro Kato, Tomohiko Matsuura, Mitsugu Kanehira, Ryo Takata, Wataru Obara

**Affiliations:** Department of Urology, Iwate Medical University School of Medicine, 19-1 Uchimaru, Morioka-shi, Iwate, 020-8505 Japan

**Keywords:** Sunitinib, Renal cell carcinoma, Hematotoxicity, Asian population

## Abstract

**Background:**

A high incidence of severe hematological adverse events during sunitinib treatment complicates decision making on dose and treatment cycle. We identified the characteristics of early-onset hematotoxicity of sunitinib in Japanese patients with renal cell carcinoma (RCC).

**Methods:**

Seventy-nine patients were treated with sunitinib as 6-week cycles of “4-week on 2-week off” schedule. To evaluate early-onset hematotoxicity, we compared patients with dose reduction during the first cycle (dose-reduced group, *n* = 57) and those who maintained the initial dose (dose-maintained group, *n* = 22). *ABCG2* and *FLT3* genotypes were analyzed for association between hematotoxicity and reported gene polymorphisms.

**Results:**

Mean relative dose intensity (RDI) was similar in the two groups during the first 2 weeks of dosing in the first cycle, but was significantly lower in the dose-reduced group during the last 2 weeks. Lymphocytopenia and thrombocytopenia were observed in the dose-reduced group within the first 2 weeks. Genetic analysis indicated a significantly higher frequency of *FLT3* 738 T/C polymorphism in the dose-reduced group, but no significant difference in the *ABCG2* 421 C/A polymorphism.

**Conclusions:**

This study showed a high incidence of sunitinib-induced hematotoxicity in Japanese patients with RCC, many of whom need dose adjustment during the first cycle. Further studies should verify whether dose adjustment based on early-onset thrombocytopenia prolongs sunitinib treatment.

## Background

Sunitinib, an orally administered tyrosine kinase inhibitor (TKI), has been evaluated in a randomized phase 3 trial in comparison with interferon alfa in patients with metastatic renal cell carcinoma (RCC) [1]. Their study demonstrated that sunitinib was superior to interferon alfa in prolonging progression-free survival (PFS) and maintaining quality of life. Recent studies showed that Asian populations had more sunitinib-induced adverse events than Caucasian patients [2–4]. A subgroup analysis of Japanese patients showed prolongation of PFS but more hematological adverse events [5]. A post-marketing surveillance study in Japan found that the most common adverse event was a laboratory test abnormality and that severe adverse events occurred within 4 weeks after starting sunitinib [6]. In the clinical setting, Japanese patients have difficulties maintaining the original “4-week on 2-week off” dosing regimen because of a high incidence of adverse events.

Pharmacokinetic and pharmacodynamic factors are supposed to affect the toxicity of sunitinib. A previous pharmacokinetic study has shown that ATP-binding cassette, sub-family G, member 2 (*ABCG2*) 421 C/A, the most common mutant allele among *ABCG2* polymorphisms in Japanese population [7, 8], is associated with increased exposure of sunitinib [9, 10]. Fms related tyrosine kinase 3 (*FLT3*) is a pharmacodynamic gene and one of the kinases targeted by sunitinib, and the association between the *FLT3* 738 T/C polymorphism and sunitinib-induced bone marrow toxicity has been reported [11, 12].

The aim of the present study was to evaluate the characteristics of hematological toxicity of sunitinib in Japanese patients with RCC and to identify the relationship between the relative dose intensity (RDI) during the first cycle and early-onset of hematological toxicity.

## Methods

### Patients

Seventy-nine patients treated with sunitinib between June 2008 and March 2015 at the Iwate Medical University were analyzed. All patients received 6-week cycles with a “4-weeks on 2-weeks off” schedule. During therapy, dose interruption and reduction were permitted if severe adverse event occurred. To determine RDI, sunitinib 50 mg/day with a 4-week on and 2-week off schedule was considered the planned therapy based on the Pharmaceuticals and Medical Devices Agency (PMDA)-approved dose/schedule for RCC. The institutional review board of our institution approved the present study (approval number: H26–91). Analysis of gene polymorphism was performed in patients who gave informed consent.

### Laboratory assessments

Blood samples were collected at baseline and on days 7, 14, 21, 28, and 42 during the first cycle. Bone marrow function, renal function (serum creatinine), liver function and other laboratory tests were conducted. Laboratory abnormalities were scored according to the criteria of Common Toxicity Criteria for Adverse Events (CTCAE) version 4.0. To evaluate clinical features of early-onset hematotoxicity, we compared blood and serum data in patients who required dose reduction or discontinuation during the first cycle (dose-reduced group), and patients who maintained the starting dose throughout the first cycle (dose-maintained group).

### Analysis of polymorphism

To examine the relationship between hematotoxicity and reported gene polymorphisms, 36 patients who gave informed consent were analyzed for the *ABCG2* genotype associated with high sunitinib exposure, and for the *FLT3* genotype which was reported to be related to sunitinib-induced myelosuppression.

Blood samples were collected in heparinized tubes and frozen (−80 °C) until required for assay. Probes and primers for typing each SNP by TaqMan SNP genotyping assays were synthesized by Thermo Fisher Scientific (Waltham, MA). Real-time PCR with cycle reactions of 10 min at 95 °C followed by 57 cycles of 15 s at 92 °C, 60 s at 60 s, and 5 min at 50 °C was performed for reaction mixtures containing sample DNA, probes, and primers in 96-well plates with a LightCycler 480 (F. Hoffmann-La Roche, Basel, Switzerland). SNP calls were done by an in-house software of SNP Data Viewer v3.1 (System Biotics, Sagamihara, Japan).

### Data analysis

Laboratory abnormalities graded according to CTCAE were analyzed using the Pearson test and Fisher test. Changes in laboratory data from baseline were assessed using a paired t-test or an exact paired Wilcoxon test. Fisher’s exact test or chi-square test and t-test were used to compare patient characteristics. *P* values less than 0.05 were considered statistically significant. To determine RDI, sunitinib at 50 mg/day with a 4-week on and 2-week off schedule was considered the planned therapy based on the PMDA-approved dose/schedule for RCC. Time to treatment failure (TTF) was estimated by the Kaplan-Meier method, and log-rank test was used to compare TTF between groups. *P* values less than 0.05 were considered statistically significant.

## Results

### Patients

**Fig. 1:**
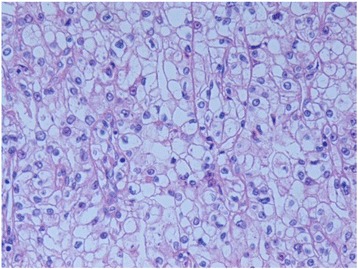
Histological clear cell subtype. Obtained from a patient who received cytoreductive nephrectomy following sunitinib therapy. Objective magnification: 400×

The subjects comprised 55 men and 24 women with a median age of 68 (range 39–82) years. Sixty-two patients had a Karnofsky performance status (KPS) rating of 80%–100% (mean 90.8% ± 1.7%) at the start of sunitinib treatment. According to the Memorial Sloan-Kettering Cancer Center (MSKCC) risk criteria [1], 23 patients (29%) were classified as poor risk. The most common sites of metastases were the lungs and lymph nodes. Forty-one patients had received a prior nephrectomy, while 5 patients received cytoreductive nephrectomy following administration of sunitinib. Histology showed clear cell RCC in 47 patients (Fig. 1) and papillary RCC in one patient. Sunitinib was given as first-line therapy in 64 patients. As a starting dose, 56 patients were administered sunitinib at 50 mg, while 23 patients were given lower doses at the discretion of clinical doctors. During the first cycle, 22 patients (28%) maintained the starting dose while 57 patients (72%) required reduction or discontinuation of sunitinib due to adverse events. The mean RDI of sunitinib for the last 2 weeks of dosing in the first cycle was significantly lower than that for the first 2 weeks (47.1% versus 84.9%, *p* < 0.01).

### Hematotoxicity

Laboratory abnormalities during the first cycle and weeks 0–2 of the first cycle are shown in Table 1. Seventy-two percent of all patients had low hemoglobin, and 61% had hypoalbuminemia before the initiation of sunitinib therapy. The most common adverse hematological events were hypoalbuminemia (95%), platelet count decreased (87%), hemoglobin decreased (85%), and Aspartate aminotransferase (AST) increased (73%) during the first cycle. Compared with the baseline, the number of all adverse events other than hyperuricemia increased significantly during therapy. The most common moderate (grade 2) and severe (grades 3 and 4) adverse events were hemoglobin decreased (54%), hypoalbuminemia (51%), and platelet count decreased (43%).

**Table 1:** Laboratory abnormalities

		All Grades n (%)	Grade 1 n (%)	Grade 2 n (%)	Grade 3/4 n (%)	*p* value
White blood cell count decreased	pretreatment	2 (3)	2 (3)	0 (0)	0 (0)	
	0-2 week of first cycle	10 (13)	4 (5)	6 (8)	0 (0)	0.10
	First cycle	44 (56)	12 (15)	27 (34)	5 (6)	<0.01
Neutrophil count decreased	pretreatment	1 (1)	1 (1)	0 (0)	0 (0)	
	0-2 week of first cycle	4 (5)	4 (5)	0 (0)	0 (0)	0.56
	First cycle	33 (42)	12 (15)	18 (23)	3 (4)	<0.01
Lymphocyte count decreased	pretreatment	22 (28)	13 (16)	7 (9)	2 (3)	
	0-2 week of first cycle	40 (51)	11 (14)	8 (10)	11 (14)	<0.01
	First cycle	46 (58)	18 (23)	12 (15)	16 (20)	<0.01
Hemoglobin decreased	pretreatment	57 (72)	34 (43)	15 (19)	8 (10)	
	0-2 week of first cycle	52 (67)	31 (39)	18 (23)	3 (4)	0.30
	First cycle	67 (85)	24 (30)	28 (35)	15 (19)	<0.01
Platelet count decreased	pretreatment	9 (11)	9 (11)	0 (0)	0 (0)	
	0-2 week of first cycle	45 (57)	35 (44)	6 (8)	4 (5)	<0.01
	First cycle	69 (87)	33 (42)	20 (25)	16 (21)	<0.01
Creatinine increased	pretreatment	29 (36)	26 (33)	2 (3)	1 (1)	
	0-2 week of first cycle	46 (58)	42 (53)	4 (5)	0 (0)	0.04
	First cycle	46 (58)	41 (52)	4 (5)	1 (1)	0.02
Aspartate aminotransferase increased	pretreatment	12 (15)	12 (15)	0 (0)	0 (0)	
	0-2 week of first cycle	42 (53)	34 (43)	6 (8)	2 (3)	<0.01
	First cycle	58 (73)	47 (59)	7 (9)	4 (5)	<0.01
Alanine aminotransferase increased	pretreatment	16 (20)	16 (20)	0 (0)	0 (0)	
	0-2 week of first cycle	33 (42)	28 (35)	4 (5)	1 (1)	<0.01
	First cycle	55 (70)	45 (57)	5 (6)	5 (6)	<0.01
Hypoalbuminemia	pretreatment	48 (61)	28 (35)	18 (23)	2 (3)	
	0-2 week of first cycle	69 (87)	33 (42)	30 (38)	6 (8)	<0.01
	First cycle	75 (95)	27 (34)	38 (48)	10 (13)	<0.01
Blood bilirubin increased	pretreatment	0 (0)	0 (0)	0 (0)	0 (0)	
	0-2 week of first cycle	3 (4)	3 (4)	0 (0)	0 (0)	0.36
	First cycle	6 (8)	5 (6)	0 (0)	1 (1)	0.02
Amylase increased	pretreatment	19 (24)	16 (20)	3 (4)	0 (0)	
	0-2 week of first cycle	29 (37)	19 (24)	8 (10)	2 (3)	0.03
	First cycle	42 (53)	20 (25)	11 (14)	11 (14)	<0.01

In all subjects, the mean dose during the last 2 weeks of dosing in the first cycle decreased significantly. Therefore, we analyzed the adverse events with early onset (within the first 2 weeks of dosing). The most common early-onset adverse hematological events were hypoalbuminemia (87%), hemoglobin decreased (67%), creatinine increased (58%), platelet count decreased (57%), AST increased (53%), and lymphocyte count decreased (51%). Among these adverse events, the incidence rate of hemoglobin decreased but was not significantly different compared with the baseline data.

### Analysis of dose-maintained and dose-reduced groups

To evaluate the clinical features of early-onset hematotoxicity related to dose reduction, we performed an analysis by dividing the patients into a dose-reduced group (*n* = 57) and a dose-maintained group (*n* = 22). Patient characteristics of the two groups are summarized in Table 2. Patient background did not differ significantly between the two groups. Analysis of patient characteristics showed an older age and lower body surface area (BSA) in the dose-reduced group than in the dose-maintained group while the starting dose was similar in the two groups. The mean RDI during the first 2 weeks of dosing was similar in the two groups (83.9% ± 2.6% versus 87.5% ± 4.2%, *p* = 0.47) although the mean RDI during the last 2 weeks was significantly lower in the dose-reduced group than in the dose-maintained group (31.0% ± 3.4% versus 88.6% ± 5.5%, *p* < 0.01).

**Table 2:** Patient characteristics of the two groups

Characteristics		Dose-reduced group	Dose-maintained group	Univariate analysis
		*n* = 57	*n* = 22	*p* value
Sex (men), n (%)		38 (66.7)	17 (77.3)	0.42
Mean age ± 2SD, year		67.2 ± 9.7	62.1 ± 10.1	0.04
Mean KPS ± 2SD, %		91.1 ± 15.4	90.5 ± 14.6	0.87
Mean BMI ± 2SD, kg/m2		21.9 ± 3.3	24.1 ± 4.5	0.05
Mean BSA ± 2SD, m2		1.6 ± 0.2	1.7 ± 0.2	<0.01
Concomitant CYP3A4 metabolized drug, n (%)		27 (47)	12 (55)	0.62
MSKCC risk score, n Favorable/Intermediate/Poor		4/36/17	2/14/6	0.94
Histological clear cell subtype, n (%)		32 (56)	15 (68)	0.77
Prior nephrectomy, n (%)		29 (51)	12 (55)	0.90
Common sites of metastasis, n (%)	Lung	29 (51)	16 (73)	0.13
	Bone	15 (26)	5 (23)	1.00
	Liver	6 (11)	2 (9)	1.00
	Lymph node	18 (32)	6 (27)	0.79
Starting dose 50/37.5/25 mg, %		72/21/7	68/14/18	0.29
RDI during the first two weeks of dosing, mean ± 2SE, %		83.9 ± 2.6	87.5 ± 4.2	0.47
RDI during the last two weeks of dosing, mean ± 2SE, %		31.0 ± 3.4	88.6 ± 5.5	<0.01

*KPS* Karnofsky performance status scale, *BMI* body mass index, *BSA* body surface area, *MSKCC* MemorialSloan–Kettering Cancer Centre, *RDI* relative dose intensity

Adverse events of the two groups are shown in Table 3. The numbers of all adverse events increased during the first cycle in both groups. On day 14 of the first cycle, the lymphocyte and platelet count decreased which occurred significantly more frequently in the dose-reduced group.

**Table 3:** Laboratory abnormalities of the two groups

		Dose-reduced group (*n* = 57)			Dose-maintained group (*n* = 22)			
		All Grades n (%)	Grade 1/2 n (%)	Grade 3/4 n (%)	All Grades n (%)	Grade 1/2 n (%)	Grade 3/4 n (%)	*p* value
White blood cell count decreased	pretreatment	2 (3)	2 (3)	0 (0)	0 (0)	0 (0)	0 (0)	1.00
	First 2 weeks	10 (18)	10 (18)	0 (0)	0 (0)	0 (0)	0 (0)	0.05
	First cycle	33 (58)	29 (51)	4 (7)	11 (50)	10 (46)	1 (5)	0.61
Neutrophil count decreased	Pretreatment	1 (2)	1 (2)	0 (0)	0 (0)	0 (0)	0 (0)	1.00
	First 2 weeks	3 (5)	3 (5)	0 (0)	1 (5)	1 (5)	0 (0)	1.00
	First cycle	26 (46)	24 (42)	2 (3)	7 (32)	6 (27)	1 (5)	0.58
Lymphocyte count decreased	pretreatment	17 (30)	15 (27)	2 (3)	5 (23)	5 (23)	0 (0)	0.58
	First 2 weeks	28 (49)	17 (30)	11 (19)	2 (9)	2 (9)	0 (0)	<0.01
	First cycle	37 (65)	21 (37)	16 (28)	9 (41)	9 (41)	0 (0)	0.13
Hemoglobin decreased	pretreatment	43 (75)	38 (67)	5 (9)	14 (65)	11 (50)	3 (14)	0.40
	First 2 weeks	38 (67)	35 (61)	3 (5)	14 (65)	14 (65)	0 (0)	0.80
	First cycle	48 (84)	36 (63)	12 (21)	2 (9)	2 (9)	0 (0)	1.00
Platelet count decreased	pretreatment	7 (12)	7 (12)	0 (0)	9 (41)	9 (41)	0 (0)	1.00
	First 2 weeks	37 (65)	34 (60)	3 (5)	8 (37)	7 (32)	1 (5)	0.03
	First cycle	50 (89)	36 (64)	14 (25)	19 (86)	17 (77)	2 (9)	1.00
Creatinine increased	pretreatment	21 (37)	20 (36)	1 (2)	8 (36)	8 (36)	0 (0)	1.00
	First 2 weeks	33 (58)	33 (58)	0 (0)	13 (59)	13 (59)	0 (0)	1.00
	First cycle	33 (58)	32 (56)	1 (2)	13 (59)	13 (59)	0 (0)	1.00
Aspartate aminotransferase increased	pretreatment	10 (18)	10 (18)	0 (0)	2 (9)	2 (9)	0 (0)	0.49
	First 2 weeks	34 (61)	32 (56)	2 (4)	8 (36)	8 (36)	0 (0)	0.08
	First cycle	45 (79)	43 (76)	2 (4)	13 (59)	11 (50)	2 (9)	0.09
Alanine aminotransferase increased	pretreatment	13 (23)	13 (23)	0 (0)	3 (14)	3 (14)	0 (0)	0.53
	First 2 weeks	26 (46)	25 (44)	1 (2)	7 (32)	7 (32)	0 (0)	0.79
	First cycle	39 (68)	36 (63)	3 (5)	16 (73)	14 (64)	2 (9)	0.89
Hypoalbuminemia	pretreatment	38 (67)	37 (65)	1 (2)	10 (45)	9 (41)	1 (5)	0.12
	First 2 weeks	52 (92)	48 (84)	4 (7)	17 (77)	15 (68)	2 (9)	0.24
	First cycle	54 (95)	46 (81)	8 (14)	21 (95)	19 (86)	2 (9)	1.00
Blood bilirubin increased	pretreatment	0 (0)	0 (0)	0 (0)	0 (0)	0 (0)	0 (0)	N.D.
	First 2 weeks	2 (4)	2 (4)	0 (0)	1 (5)	1 (5)	0 (0)	1.00
	First cycle	4 (7)	3 (5)	1 (2)	2 (9)	2 (9)	0 (0)	0.67
Amylase increased	pretreatment	15 (26)	15 (27)	0 (0)	4 (18)	4 (18)	0 (0)	0.57
	First 2 weeks	23 (40)	21 (37)	2 (4)	6 (27)	6 (27)	0 (0)	0.42
	First cycle	33 (58)	23 (41)	10 (18)	9 (41)	8 (36)	1 (5)	0.20
Hyperuricemia	pretreatment	10 (18)	10 (18)	0 (0)	6 (27)	6 (27)	0 (0)	0.54
	First 2 weeks	14 (25)	14 (25)	0 (0)	5 (23)	5 (23)	0 (0)	1.00
	First cycle	14 (25)	14 (25)	0 (0)	7 (32)	7 (32)	0 (0)	0.57
Hypophosphatemia	pretreatment	4 (7)	2 (4)	2 (4)	3 (14)	2 (9)	1 (5)	0.40
	First 2 weeks	10 (18)	8 (15)	2 (4)	0 (0)	0 (0)	0 (0)	0.05
	First cycle	26 (46)	21 (37)	5 (9)	8 (36)	7 (32)	1 (5)	0.60

Data are expressed in number of cases, with percentage in parenthesis

Laboratory data of lymphocyte and platelet counts in the two groups are shown in Table 4. The mean lymphocyte count in the dose-reduced group was significantly lower than in the dose-maintained group on day 14 (887/m^2^ versus 1405/m^2^, *p* < 0.01), but increased gradually to a similar level by the end of the first cycle.

**Table 4:** Laboratory data during the first cycle

	Group	Baseline	p	Day 7	p	Day 14	p	Day 21	p	Day 28	p	Day 42	p
Lymphocyte count ×10^3^ /μL	Dose-reduced	1.2 ± 0.6	0.08	1.3 ± 0.1	0.14	0.9 ± 0.1	<0.01	0.9 ± 0.1	0.17	1.1 ± 0.1	0.17	1.2 ± 0.1	0.68
	Dose-maintained	1.4 ± 0.1		1.6 ± 0.1		1.4 ± 0.1		1.2 ± 0.2		1.2 ± 0.1		1.3 ± 0.1	
Platelet count ×10^4^ /μL	Dose-reduced	30.1 ± 2.0	0.80	26.8 ± 2.0	0.89	15.9 ± 1.2	0.04	12.0 ± 1.4	0.17	16.8 ± 1.8	0.01	24.1 ± 1.9	0.28
	Dose-maintained	30.9 ± 2.6		27.3 ± 3.1		22.0 ± 2.4		15.6 ± 2.1		11.6 ± 1.0		20.8 ± 2.3	

Data are expressed as mean ± SE.

The mean platelet count in the dose-reduced group was significantly lower on day 14 (15.9 × 10^4^/m^2^ versus 22.0 × 10^4^/m^2^, *p* < 0.01), but recovered gradually following dose reduction or discontinuation. On the other hand, the platelet count in the dose-maintained group decreased gradually and was significantly lower than in the dose-reduced group on day 28 (11.6 × 10^4^/m^2^ versus 16.8 × 10^4^/m^2^, *p* = 0.01).

Thirty-three patients in the dose-reduced group continued sunitinib treatment for the second cycle. The mean RDI during the whole second cycle was similar to that during the first cycle (61.2% versus 65.9%, *p* = 0.26). The mean RDI for the last 2 weeks of dosing in the second cycle was significantly higher than that in the first cycle (57.5% versus 43.1%, *p* = 0.02). In these patients, the mean thrombocyte count during the second cycle was stable (first 2 weeks; 22.5 × 10^4^/m^2^, last 2 weeks; 22.0 × 10^4^/m^2^, *p* = 0.81).

We also conducted analysis of patients with clear cell RCC. Patients with clear cell RCC were divided into a dose-reduced group (*n* = 32) and a dose-maintained group (*n* = 15). The patient background did not differ significantly between the two groups. In both groups, approximately 80% of patients received a prior nephrectomy. The mean RDI during the first 2 weeks of dosing was similar in the two groups (86.7% ± 3.1% versus 93.3% ± 3.8%, *p* = 0.19) although the mean RDI during the last 2 weeks was significantly lower in the dose-reduced group than in the dose-maintained group (30.8% ± 5.1% versus 95.0% ± 2.7%, *p* < 0.01).

The numbers of all adverse events increased during the first cycle in both groups. However, the number of adverse events showed no significant difference between the two groups (data not shown). The mean lymphocyte count in the dose-reduced group tended to be lower than in the dose-maintained group on day 14 (900/m^2^ versus 1500/m^2^, *p* = 0.05). Mean platelet count in the dose-reduced group tended to be lower on day 14 (16.4 × 10^4^/m^2^ versus 23.4 × 10^4^/m^2^, *p* = 0.09), but recovered gradually following dose reduction or discontinuation. On the other hand, platelet count in the dose-maintained group decreased gradually and tended to be lower than in the dose-reduced group on day 28 (16.6 × 10^4^/m^2^ versus 11.7 × 10^4^/m^2^, *p* = 0.06).

### Analysis of ABCG2 and FLT3 polymorphism

To investigate the relationship between hematotoxicity and gene polymorphisms associated with sunitinib, we analyzed two genotypes in 36 patients who gave informed consent. Genetic analysis for the *ABCG2* polymorphism and *FLT3* polymorphism in the two groups are shown in Table 5. Among 36 patients, 25 patients (69%) were in the dose-reduced group, and 11 (31%) in the dose-maintained group. Genetic analysis found no significant difference in *ABCG2* 421 C/A polymorphism between the two groups (dose-reduced group: wild-type allele *n* = 12, variant allele *n* = 13; dose-maintained group: wild-type allele *n* = 6, variant allele *n* = 5; *p* = 1.00). We analyzed laboratory data related to *ABCG2* polymorphism (Table 6). The mean lymphocyte count of patients with the *ABCG2* variant allele tended to be lower than those with the wild-type allele on day 14 (700/m^2^ versus 1200/m^2^, *p* = 0.08). The mean platelet count showed no significant difference between the two groups on day 14 (13.5 × 10^4^/m^2^ versus 17.9 × 10^4^/m^2^, *p* = 0.24). The lactate dehydrogenase (LDH) level in the group with *ABCG2* variant allele was significantly higher than wild-type allele group on day 14 (537.7 IU/L versus 324.5 IU/L, *p* = 0.04). Among those with the variant allele, 2 patients with the *ABCG2* 421 genotype homozygous for the A allele required dose reduction in the first cycle. One was treated with a starting dose of 50 mg/day. He later developed a high fever, grade 2 leukopenia, and grade 3 thrombocytopenia in the second week of the first cycle. The other patient was treated at a starting dose of 25 mg/day. She developed grade 2 thrombocytopenia in the third week of the first cycle.

**Table 5:** Genetic analysis for the two groups

Genotypes		Dose-reduced group	Dose-maintained group	Univariate analysis
		*n* = 25	*n* = 11	*p* value
ABCG2 421 C/A, n (%)	Wild-type allele	12 (67)	6 (33)	1.00
	Variant allele	13 (72)	5 (28)	
FLT3 738 T/C, n (%)	Wild-type allele	11 (55)	9 (45)	0.04
	Variant allele	14 (88)	2 (12)

**Table 6:** Laboratory data during the first cycle related to *ABCG2* and *FLT3* genotypes

	Genotype		Baseline	p	Day 7	p	Day 14	p	Day 28	p	Day 42	p
Lymphocyte count ×10^3^ /μL	ABCG2 421 C/A	Wild-type	1.3 ± 0.1	0.88	1.6 ± 0.2	0.22	1.2 ± 0.2	0.08	1.3 ± 0.1	0.24	1.3 ± 0.1	0.57
		Variant	1.4 ± 0.1		1.3 ± 0.2		0.7 ± 0.1		1.1 ± 0.1		1.2 ± 0.1	
	FLT3 738 T/C	Wild-type	1.2 ± 0.1	0.59	1.4 ± 0.1	0.98	1.1 ± 0.2	0.20	1.1 ± 0.1	0.31	1.1 ± 0.1	0.16
		Variant	1.3 ± 0.1		1.4 ± 0.2		0.8 ± 0.1		1.3 ± 0.2		1.4 ± 0.1	
Platelet count ×10^4^ /μL	ABCG2 421 C/A	Wild-type	29.5 ± 4.0	0.78	23.5 ± 3.3	0.78	17.9 ± 3.0	0.24	16.2 ± 2.7	0.99	23.5 ± 2.6	0.34
		Variant	28.4 ± 1.3		24.6 ± 1.8		13.5 ± 2.2		16.1 ± 2.7		20.4 ± 1.7	
	FLT3 738 T/C	Wild-type	30.3 ± 3.0	0.40	26.2 ± 2.8	0.16	18.9 ± 3.0	0.04	16.3 ± 2.9	0.99	21.4 ± 2.4	0.66
		Variant	27.1 ± 2.4		21.0 ± 2.3		11.7 ± 1.5		16.0 ± 2.4		22.7 ± 1.9	
Aspartate aminotransferase IU/L	ABCG2 421 C/A	Wild-type	32.7 ± 4.6	0.10	42.0 ± 8.9	0.28	45.1 ± 6.9	0.23	36.3 ± 4.4	0.47	28.7 ± 3.4	0.33
		Variant	23.8 ± 2.4		31.6 ± 2.6		63.7 ± 13.7		31.6 ± 4.6		24.4 ± 2.7	
	FLT3 738 T/C	Wild-type	27.4 ± 3.9	0.71	38.1 ± 7.6	0.72	38.7 ± 4.6	0.04	35.0 ± 4.6	0.72	24.7 ± 2.4	0.36
		Variant	29.4 ± 3.5		35.0 ± 3.5		73.6 ± 15.2		32.7 ± 4.4		28.9 ± 3.9	
Lactate dehydrogenase IU/L	ABCG2 421 C/A	Wild-type	223.6 ± 17.1	0.88	263.9 ± 27.9	0.36	324.5 ± 32.2	0.04	270.7 ± 20.0	0.47	229.1 ± 14.4	0.96
		Variant	228.6 ± 27.4		315.5 ± 47.8		537.7 ± 90.9		295.9 ± 27.9		227.9 ± 17.5	
	FLT3 738 T/C	Wild-type	221.8 ± 15.6	0.78	286.7 ± 39.1	0.90	422.9 ± 85.0	0.81	287.6 ± 19.8	0.79	235.4 ± 15.5	0.50
		Variant	231.4 ± 30.6		294.0 ± 38.8		447.4 ± 55.3		277.8 ± 30.2		220.0 ± 16.2	
Alkaline phosphatase IU/L	ABCG2 421 C/A	Wild-type	462.5 ± 128.4	0.84	520.7 ± 207.3	0.65	342.3 ± 33.9	0.35	504.3 ± 119.8	0.73	415.4 ± 92.3	0.87
		Variant	426.4 ± 121.6		418.0 ± 87.2		412.4 ± 65.4		452.8 ± 88.3		440.7 ± 120.8	
	FLT3 738 T/C	Wild-type	422.4 ± 117.3	0.78	509.4 ± 194.3	0.70	299.7 ± 19.7	0.03	466.6 ± 114.2	0.87	384.4 ± 89.8	0.54
		Variant	472.1 ± 134.4		423.9 ± 93.7		454.9 ± 66.2		490.3 ± 79.7		479.1 ± 124.9	

Data are expressed as mean ± SE.

The frequency of the *FLT3* 738 T/C polymorphism was significantly higher in the dose-reduced group (wild-type allele *n* = 11, heterozygous variant allele *n* = 14) than in the dose-maintained group (wild-type *n* = 9, heterozygous variant allele *n* = 2, *p* = 0.04). We analyzed laboratory data related to *FLT3* polymorphism (Table 6). The mean platelet count of patients with the *FLT3* variant allele was significantly lower than wild-type allele on day 14 (11.7 × 10^4^/m^2^ versus 18.9 × 10^4^/m^2^, *p* = 0.04). The mean lymphocyte count showed no significant relation to the *FLT3* genotypes on day 14 (800/m^2^ versus 1100/m^2^, *p* = 0.20). The Asparate aminotransferase (AST) and Alkaline phosphatase (ALP) of patients with *FLT3* variant allele were significantly higher than wild-type allele on day 14 (73.6 IU/L versus 38.7 IU/L, *p* = 0.04, 454.9 IU/L versus 299.7 IU/L, *p* = 0.03, respectively). Two patients in the dose-maintained group had the *FLT3* 738 C-allele. Both patients were given 50 mg/day during the first cycle. One was a 40 year-old obese (Body Mass Index 33 kg/m2) woman. She had grade 2 thrombocytopenia during the first cycle and grade 3 thrombocytopenia at the end of the second cycle, when sunitinib was then discontinued. The other patient was a 56 year-old man. He had grade 1 thrombocytopenia on day 14 of the first cycle and continued sunitinib therapy.

### Efficacy

The median follow-up period was 15.7 (range 0.9 to 78.7) months. The median TTF in all patients was estimated to be 7.0 (range 0.1 to 37.6) months. The most common reason for discontinuation was adverse events (51%). The TTF tended to be shorter in the dose-reduced group than in the dose-maintained group (6.9 months versus 9.0 months, *p* = 0.23). The median TTF in patients who had early-onset thrombocytopenia tended to be longer than those without early-onset thrombocytopenia in either group (data not shown). The median TTF according to the gene polymorphism in both *ABCG2* and *FLT3* showed no significant differences.

## Discussion

In the present study, we retrospectively analyzed sunitinib-induced early-onset hematotoxicity. Analysis of hematological adverse events during the first cycle in all patients showed a high incidence of hypoalbuminemia, thrombocytopenia, anemia, and elevated AST. During the first 2 weeks of the first cycle, the most common adverse events were hypoalbuminemia, anemia, increased creatinine, and thrombocytopenia. An expanded access trial reported thrombocytopenia (all grade 22%, grade 3/4 8%), anemia (all grade 15%, grade 3/4 4%), and neutropenia (all grade 15%, grade 3/4 6%) as common hematological adverse events [13]. A Japanese subgroup analysis showed a high incidence of thrombocytopenia (first-line population and pretreated population: 96% and 88%, respectively), leukopenia (88% and 85%), and lymphocytopenia (76% and 58%) [5]. The incidence of thrombocytopenia in our subjects was the same as that reported by a Japanese subgroup analysis [5], while the incidence of hypoalbuminemia and anemia was higher. A possible reason is the higher proportion of poor risk patients in our study than in previous studies [1, 13], since 60–70% of our patients had anemia and/or hypoalbuminemia prior to sunitinib treatment.

Generally, dose adjustment for chemotherapeutic drugs is decided based on several factors including BSA, renal function, and liver function. However, for molecular-targeted agents, there are no established criteria to decide the starting and maintenance doses. In the clinical setting, the starting dose is decided by the attending physician considering patient characteristics including sex, performance status, and physical constitution, and sometimes requires adjustment because of hematotoxicity or other toxicities. Previous reports suggest that the RDI of the whole cycle affects efficacy and toxicity of sunitinib [14, 15]. Japanese patients have difficulties maintaining the starting sunitinib dose during the first cycle, and a few reports described dose changes during the dosing period. In our institute, the most common reason for dose reduction or discontinuation during the first cycle is hematotoxicity. In our subjects, sunitinib dose in the last 2 weeks of the first cycle was significantly reduced compared with that in the first 2 weeks. Increased exposure to sunitinib is supposed to increase the incidence or severity of hematotoxicity. Therefore, our study focused on the group that developed early-onset hematological adverse events and required dose reduction. The dose-reduced group was older with a lower BSA than the dose-maintained group, but the starting dose was similar in both groups. The meta-analysis conducted by Houk et al. [16] suggests that individual covariates (race, gender, body weight, and ECOG Performance status score) have minimal effects on the pharmacokinetics of sunitinib and its primary metabolite (SU12662). The early-onset hematological adverse events in the dose-reduced group were lymphocytopenia and thrombocytopenia. In this group, lymphocyte and thrombocyte counts decreased in the second to third week and took a few weeks to recover to the baseline level. In the dose-maintained group, however, thrombocyte counts decreased gradually during dosing and were significantly lower than that in the dose-reduced group on day 28. Previous studies reported a pharmacogenetic association with hematological adverse events on day 28 or day 42 of the treatment cycle [11, 12]. Our result suggests that laboratory data on day 28 or day 42 may include not only the lowest levels in patients who do not need dose reduction or discontinuation, but also the recovered levels after the dose has been modified according to toxicity.

Subgroup analysis in patients with clear cell RCC showed no significant difference in the incidence of hematotoxicity between the dose-reduced group and the dose-maintained group. In this study, approximately 80% of patients with clear cell histology have previously undergone a nephrectomy. Thirty-one patients started sunitinib treatment in the diagnosis of imaging modality without histological diagnosis. Patients with undetermined histology tended to have a lower KPS and a higher rate of MSKCC poor risk than patients with clear cell RCC [KPS: 88.4% versus 92.3%, MSKCC poor risk group: *n* = 13 (42%) versus 10 (21%)]. Surgery or biopsy is needed to evaluate histological subtype although biopsy would be a relatively invasive procedure for patients. We assumed that the physicians might avoid biopsy particularly in patients with low performance status due to disease progression or comorbidities. We considered that patients with undetermined histology might include patients with advanced clear cell RCC and result of analysis of clear cell RCC subgroup would include selective bias.

In this study, 33 patients in the dose-reduced group continued sunitinib treatment in the second cycle. Although the starting dose for the second cycle was reduced, a higher dose compared to the first cycle was maintained for the last 2 weeks during the second cycle. In these patients, the mean thrombocyte count during the second cycle was stable (225.0 × 10^4^/m^2^ during the first 2 weeks, and 220.3 × 10^4^/m^2^ during the last 2 weeks, *p* = 0.81). These results indicate that dose adjustment based on early-onset hematotoxicity in the first cycle avoids treatment discontinuation. Appropriate dose modification during the early cycles of sunitinib treatment is potentially useful in identifying sunitinib-resistant patients who need alternative TKI therapy or a new therapy with different mechanisms of action such as the mammalian target of rapamycin (mTOR) inhibitor, or sunitinib-intolerant patients who require therapy with different adverse event profiles.

Our analysis showed a high incidence of hematological adverse events compared with non-Asian populations [1, 13], with characteristic changes in lymphocyte and thrombocyte counts in the early treatment period. Based on these results, we analyzed the *ABCG2* genotype which is related to a high exposure of sunitinib in the Asian population, and the *FLT3* genotype which is associated with sunitinib-induced myelosuppression. The rate of *ABCG2* 421 C/A polymorphism showed no significant difference between the dose-reduced group and dose-maintained group. Analysis of the laboratory data of those with the *ABCG2* gene polymorphism showed that the early-onset of lymphocytopenia and elevated LDH tend to be seen in patients with the *ABCG2* variant allele, but not thrombocytopenia. Among patients having the *ABCG2* 421 A-allele, two patients with a homozygous genotype required dose reduction during the first cycle. Both patients developed moderate to severe hematological adverse events. The frequency of the *ABCG2* 421 AA genotype is 7% and that of *ABCG2* 421 CA is 39% in Japanese population [17]. Mizuno et al. [9] demonstrated the association of plasma sunitinib concentration with the intestinal absorption process, and suggested that the *ABCG2* 421 A-allele is related to increased sunitinib exposure. Miura et al. [10] reported a patient with the *ABCG2* 421 AA genotype who had a high concentration of sunitinib on day 14 of the first cycle and severe adverse events including thrombocytopenia. Our study found no relationship between the *ABCG2* genotypes and thrombocytopenia. A possible reason may be the small number of patients with homozygous alleles. Furthermore, unlike previous studies, our study was not designed to examine the relationship between the *ABCG2* genotype with pharmacokinetics, and hence may result in selection bias. The frequency of the *FLT3* 738 C-allele carriers was significantly higher in the dose-reduced group than in the dose-maintained group. Kumar et al. [11] investigated kinase selectivity and the effect on cell proliferation of the three TKIs (sunitinib, sorafenib, and pazopanib), and suggested that the strong activity of sunitinib against c-kit and *FLT3* may provide a plausible explanation for the high incidence of myelosuppression observed in clinical treatment. Van Erp et al. [12] investigated the relationship between the *FLT3* 738 genotype and thrombocyte count during the 4 weeks of the first cycle, and showed a significant decrease in thrombocyte count in the *FLT3* 738 C-allele carriers compared with *FLT3* TT individuals. Their study showed approximately 30% of patients developed thrombocytopenia during the first cycle, which was much lower than that in the Asian population. In the present study, the thrombocyte count on day 28 of the first cycle was significantly lower in the dose-maintained group than in the dose-reduced group, consistent with a previous report [12]. The thrombocyte count on day 14 of the first cycle was significant lower in the dose-reduced group than in the dose-maintained group. Analysis of laboratory data in those patients with the *FLT3* gene polymorphism showed that the thrombocyte count on day 14 was significantly lower than in patients with the wild-type allele. We also analyzed the laboratory data and SNPs of three patients who were treated with a modified schedule of “2 weeks on and 1 week off.” The mean thrombocyte count on day 14 of the two patients with the *FLT3* 738 TC (11.2 × 10^4^/m^2^) was lower than that in one patient with *FLT3* 738 TT (15.3 × 10^4^/m^2^). One of the 2 patients having the *FLT3* 738 C-allele in the dose-maintained group was a 40 year-old obese woman. She had severe thrombocytopenia at the end of the second cycle, and sunitinib was discontinued. Increased clearance with increasing body weight was reported for TKIs [18], and a previous report evaluated the effect of body weight on the pharmacokinetics of sunitinib [12]. We speculate that *FLT3* 738 C-allele carriers tend to be more susceptible to early-onset thrombocytopenia and require dose reduction. Profiles of early-onset of hematotoxicity related to gene polymorphism were different between *ABCG2* and *FLT3* genotypes. We assumed that this difference might occur due to pharmacokinetics effects related to *ABCG2* polymorphism and the pharmacodynamics effects related to *FLT3*. We recommend that monitoring of laboratory data during the early dosing period is required to analyze the relationship between hematotoxicity with gene polymorphism, especially in Asian populations. Future studies should evaluate whether there is an association between these two SNPs and non-hematological adverse events such as hypertension and fatigue which can be a major problem in the clinical setting.

The median TTF of all patients was estimated to be 7.0 months. The most common reason for discontinuation of sunitinib treatment was adverse events. TTF in the dose-reduced group tended to be shorter compared to the dose-maintained group. The median TTF tended to be longer in patients who developed early-onset thrombocytopenia than in those without thrombocytopenia (data not shown). A previous study reported thrombocytopenia as a predictive biomarker of prolonged survival [19]. In the present retrospective study, discontinuation of sunitinib treatment was decided by the clinical doctors. Together with the small number of patients studied, selection bias may have influenced the result of TTF. Nevertheless, we propose that patients with early-onset thrombocytopenia should undergo dose reduction or a drug holiday, and continue treatment with adjusted doses, which may prolong the TTF. A prospective study with RDI analysis is warranted to determine the efficacy of treatment strategy with dose modification using thrombocytopenia as a biomarker. Though *ABCG2* and *FLT3* genotypes were not identified as prognostic factors in this study, dose adjustment based on these specific SNPs may prolong the period of sunitinib treatment.

Our study has several limitations. First, it was a retrospective study with a small number of cases and potential selection bias. Second, this study did not assess the plasma concentration of sunitinib, and hence lacks pharmacokinetics data. Third, not all the patients underwent SNP analysis because some patients were lost to follow-up or refused genetic analysis.

## Conclusions

In conclusion, our study showed that many patients need dose reduction during the first cycle of sunitinib, as these patients developed thrombocytopenia and lymphocytopenia during the early period of the first cycle. We consider that dose adjustment based on early-onset toxicity may prolong the period of sunitinib treatment. Further studies with longer follow-up are warranted to verify whether sunitinib dose adjustment based on early-onset toxicity improves the prognosis for RCC.

## Abbreviations

ABCG2: ATP-binding cassette sub-family G member 2
ALP: Alkaline phosphatase
AST: Aspartate aminotransferase
BSA: Body surface area
CTCAE: Common Toxicity Criteria for Adverse Events
ECOG: Eastern Cooperative Oncology Group
FLT3: Fms related tyrosine kinase 3
KPS: Karnofsky performance status
LDH: Lactate dehydrogenase
MSKCC: Memorial Sloan-Kettering Cancer Center
mTOR: Mammalian target of rapamycin
PMDA: Pharmaceuticals and Medical Devices Agency
RCC: Renal cell carcinoma
RDI: Relative dose intensity
TKI: Tyrosine kinase inhibitor
